# Influence of feeding time on daily rhythms of locomotor activity, clock genes, and epigenetic mechanisms in the liver and hypothalamus of the European sea bass (*Dicentrarchus labrax*)

**DOI:** 10.1007/s10695-025-01461-7

**Published:** 2025-02-13

**Authors:** Elisa Samorì, Inmaculada Rodríguez, José Antonio Oliver, Francisco Javier Sánchez-Vázquez, José Fernando López-Olmeda

**Affiliations:** Department of Physiology, Faculty of Biology, University of Murcia, 30100 Murcia, Spain

**Keywords:** Central and peripheral pacemakers, Clock genes, Methylation, Acetylation, Methylation potential

## Abstract

The circadian system plays a crucial role in most physiological processes. The molecular clock is linked to epigenetic mechanisms, both of which are influenced by nutrient status and, consequently, to feeding. This research investigated how feeding times (mid-light, ML, vs. mid-dark, MD) synchronize daily rhythms of behavior, clock genes, and epigenetic mechanisms in the European sea bass (*Dicentrarchus labrax*), focusing on hypothalamus and liver to assess the impact on central and peripheral pacemakers. Feeding at MD influenced the molecular clock of the hypothalamus, causing shifts in acrophases (peaks) for genes of the negative loop (*per1b*, *per2*, *cry1a*). In the liver, the ML fed group showed rhythmic expression for all clock genes, whereas only *per2* maintained the rhythms in the MD group. Epigenetic genes related to methylation (*dnmt1*, *dnmt3a*) and demethylation (*tet2*, *gadd45aa*, *mbd4*) in the liver displayed rhythmic expression in the ML group, but only *dnmt3a* maintained the rhythm in the MD group. Nutrient-related factors (SAM and SAH) showed differences between day and night, suggesting a different utilization based on feeding times. Finally, *sirt1*, a gene involved in deacetylation, displayed a clear daily rhythm in the ML group. All epigenetic genes peaked during the night (resting phase). Overall, these findings indicated feeding time serves as a potent *zeitgeber*, synchronizing circadian clock and epigenetic rhythms in the liver, with peaks during the resting phase, suggesting this phase represents the adequate time for epigenetic modifications.

**Supplementary Information:**

The online version contains supplementary material available at 10.1007/s10695-025-01461-7.

## Introduction

Living organisms present biological rhythms in their functions or processes at various levels, ranging from behavioral to molecular. These rhythms are synchronized to different external time cues, known as *zeitgebers*, such us the light–dark (LD) cycle (Vatine et al. 2011; Paredes et al. 2015; Steindal and Whitmore 2020), feeding (Lewis et al. 2020; López-Olmeda 2017) and temperature (Rensing and Ruoff 2002; Lahiri et al. 2005 and 2014; López-Olmeda and Sánchez-Vázquez 2009). In this context, the pacemakers represent functional anatomic regions capable of integrating these external stimuli, generating oscillations and transmitting them to output pathways, thereby generating overt rhythms (Pando and Sassone-Corsi 2002). In fish, the circadian system appears to be composed of a network of pacemakers present in most tissues and cells (Whitmore et al. 2000), lacking the hierarchical organization that is a distinctive feature of mammals, where the master pacemaker is located in the suprachiasmatic nucleus (SCN) (Mohawk et al. 2012).

The generation of all these rhythms relies on the molecular machinery within cells. The core molecular system of the pacemakers is referred to as the molecular clock and exhibits a fundamentally similar organization in both central and peripheral tissues of mammals and fish (Kumar and Sharma 2018). The molecular clock operates through positive and negative feedback loops that confer pacemakers their self-sustaining activity and drive the development of rhythmicity. The positive loop initiates with the transcription of *clock* and *bmal* genes, whose proteins form a heterodimer functioning as a transcription factor binding to E-box domains in the promoter regions of many genes. Among these genes, clock and Bmal promote the transcription of *per* and *cry*, which constitute the negative loop responsible for the downregulation of *clock* and *bmal* expression (Pando and Sassone-Corsi 2002; Mohawk et al. 2012; Kumar and Sharma 2018). The circadian molecular clock is regulated at various levels, relying on E-box binding domains (Hardin 2004), post-transcriptional modifications (Mehra et al. 2009), and epigenetic regulation (Eckel-Mahan and Sassone-Corsi 2013; Satou et al. 2013). The circadian system and epigenetic mechanisms are closely related. In recent years, the existence of a bidirectional pathway between these two systems has been proposed in mammals, although it remains controversial which one controls the other (Satou et al. 2013; Xia et al. 2015; Stevenson 2018). Among the epigenetic systems, DNA methylation and histone acetylation/deacetylation have been reported to be associated with the circadian system (Doi et al. 2006; Nakahata et al. 2008; Satou et al. 2013; Stevenson 2018). DNA methylation involves the addition of a methyl group to a cytosine followed by a guanine (CpG dinucleotide), particularly in CpG islands. This process facilitates DNA silencing by preventing transcription factor binding and recruiting methyl-binding proteins that act as inhibitory elements (Bird et al. 2002; Moore et al. 2013). The components involved in silencing belong to the DNA methyltransferase (Dnmts) family enzyme, which includes enzymes responsible for maintaining methylation after DNA duplication (Dnmt1) and those that promote de novo methylation (Dnmt3s) (Jurkowska et al. 2011). All genes involved in DNA methylation are S-adenosyl methionine-dependent, utilizing the methyl group produced in the conversion of S-adenosyl methionine (SAM) into S-adenosyl homocysteine (SAH) and adenine. SAH itself serves as an important inhibitor of most methyltransferases, and the SAM/SAH ratio is used to describe methylation potential (Mirbahai et al. 2013; Xia et al. 2015).

The establishment of a methylation pattern represents a crucial aspect for the cell. The methylation process is dynamic and reversible, as it involves multiple mechanisms for active removal. Demethylation can occur through oxidative and repair-based mechanisms. In the oxidative pathway, the ten-eleven-translocation enzyme (Tet) successively hydroxylates 5-methylcytosine (5mC) to 5-hydroxymethylcytosine (5hmC), followed by 5-formylcytosine (5fC) and 5-carboxylcytosine (5caC) (Tahiliani et al. 2009; Ito et al. 2010). Subsequently, 5caC can be excised by thymine-DNA glycosylase (Tdg) and replaced with an unmodified cytosine through base excision repair (BER) (He et al. 2011; Shen et al. 2013). Simultaneously, Growth Arrest and DNA-damage-inducible Protein 45 (Gadd45a) can interact with Tdg to facilitate its recruitment to target loci for subsequent excision (Niehrs & Schäfer 2012; Li et al. 2015). Gadd45aa can also interact with the deaminase Apobec (Apolipoprotein B RNA-editing catalytic component) and Mbd4 (methyl-CpG-binding domain protein 4), a BER-specific thymine glycosylase, in a coupled mechanism where the deaminase converts 5-mC to thymine, followed by final base excision of the T:G mismatch by Mbd4 (Rai et al. 2008).

Recently, the interplay between DNA methylation and the circadian system has garnered attention to elucidate the complexity and regulation of biological rhythms. This interest starts from the observation that *Dnmt3a* harbors a high-density Bmal1-binding site on its promoter, potentially rendering it a target for Bmal1 (Stevenson 2018). Moreover, *Dnmt3a/3b* may be implicated in the methylation of *Bmal1*'s promoter in various diseases, leading to its silencing (Satou et al. 2013). Furthermore, DNA methylation is not the sole epigenetic process associated with the clock machinery; histone deacetylation also plays a role. *Clock* possesses histone acetyltransferase (HAT) activity, which facilitates histone acetylation, thereby initiating the negative circuit (Doi et al. 2006). To counterbalance this activity, the histone deacetylase SIRT1 is crucial, as it targets *Clock*'s HAT action preferentially (Nakahata et al. 2008, 2009). Moreover, SIRT1 relies on NAD + as a cofactor (Suave et al. 2006), making histone deacetylation depending upon the cell's energy status. Additionally, the function of Dnmts enzymes is dependent on the availability of the amino acid methionine, which serves as a methyl donor following its conversion to SAM (Niculescu and Zeisel 2002). Consequently, feeding patterns and nutritional status exert significant influence on epigenetic regulation, a phenomenon observed in fish as well (Skjaerven et al. 2018).

Regarding feeding, food provided in a periodic manner can also serve as a potent synchronizer for fish, influencing food entrainable oscillators (FEOs) and clock gene expression, particularly in peripheral pacemakers (Lopez-Olmeda et al. 2010; Feliciano et al. 2011; Vera et al. 2013; Costa et al. 2016; Gómez-Boronat et al. 2018). Also, observable rhythms such as the daily patterns of locomotor activity, is strongly affected by feeding time, as reported in different research (Lopez-Olmeda et al. 2010; Vera et al. 2013; Gómez-Boronat et al. 2018). The daily rhythm of clock genes and their regulation by LD cycles and feeding time has been described in various fish species, both in central and peripheral tissues (López-Olmeda et al. 2010; Feliciano et al. 2011; Nisembaum et al. 2012; Vera et al. 2013), including the European sea bass (*Dicentrarchus labrax*) (Sánchez et al. 2010; Del Pozo et al. 2012; Herrero and Lepesant 2014). However, research on the presence of rhythms in epigenetic processes in fish is limited, with only a single study on zebrafish (*Danio rerio*) demonstrating that genes involved in methylation exhibit a rhythm with peak values mainly occurring during the night phase (Paredes et al. 2018). Therefore, although the circadian clock and epigenetic mechanisms appear to be interconnected at various levels in mammals, the situation in fish models, as well as the influence of feeding time, remains largely unknown.

The aim of this research was to investigate the interplay between daily rhythms of genes involved in the circadian system and epigenetic mechanisms, as well as the effect of different feeding schedules in the liver of the European sea bass. The study focuses on the liver due to its role as one of the main organs involved in nutrient processing and the presence of a food entrainable oscillator in this tissue. In addition, we studied how feeding time influences the clock genes in the hypothalamus, as a tissue primarily regulated by light. In order to deepen into the role of nutrient utilization, we also tested SAM, SAH, and the methylation potential. Finally, locomotor activity was recorded to elucidate if the appearance of the rhythm at the molecular level is linked to the phase of activity.

## Materials and methods

The experiments were conducted at the Aquaculture Laboratory of the University of Murcia, located within the Naval Base of Algameca (E.N.A., Cartagena, Spain). The experimental design followed the European Union guidelines (2010/63/UE) and the Spanish legislation (RD 53/2013 and Law 32/2007) regarding the use of laboratory animals. Approval for the study was obtained from the Committee on Ethics and Animal Welfare of the University of Murcia and the Government of the *Región de Murcia* (license number A13191003).

### Animals and housing

Juvenile European sea bass specimens (*N* = 100) were obtained from CULMAREX (Guardamar del Segura, Alicante, Spain) and divided into two 500-L tanks (50 fish/tank). The tanks are part of an open system that supplies the water from the environment. The system is equipped with biological and mechanical filters, and UV lamp that sterilizes the water before entering the system, which after passing through the different tanks, is then reinserted into the environment again. A commercial diet (Alterna Marine, Skettring, Burgos, Spain) was used for this stock fish during the acclimation, and it was provided from the operator by hand ad libitum*.* Thus, fish were fed to satiety, until fish stopped eating, during this period. The photoperiod was controlled to simulate the seasonal variation by means of a timer connected to the lights (Data Micro, Orbis, Madrid, Spain) and light onset set at *Zeitgeber* time 0 (ZT 0 h) (Espirito Santo et al. 2020). LED white lights that covered the whole visible spectrum (Suppl. Figure 1) were employed to grant illumination, with an intensity of 200 lx and an irradiance of 2.76 μmol photon/m^2^s at the water surface. Water temperature was monitored for all the experiment (HOBO PENDANT Onset Computer Corporation, MA, USA) and mirrored that of the natural environment.

### Experimental design

After 1 month of acclimation, 98 animals (47.3 ± 0.59 g body weight, mean ± SEM) were randomly divided into 14 tanks (7 fish/tank) and two groups of 7 tanks each were set considering the feeding time: one group was fed around the middle of the light phase (ML, ZT 5 h) and the second group was fed around the middle of the dark phase (MD, ZT 17 h). All tanks were fed daily with the 1% of the biomass of the fish (D2 Optibream 2P, Skettring, Burgos, Spain) and the feed ration was provided by automatic feeders (Eheim GmbH & Co. KG, model 3581, Deizisau, Germany) set to provide half of the ration (0.5% of the body weight) 30 min before ML or MD, depending on the group, and the other half 30 min after ML or MD, in order to optimize feed consumption and reduce waste. Fish were kept under these conditions for 30 days. During this period, locomotor activity was continuously recorded by means of infrared photocells (Omron, mod E3S-AD62, Kyoto, Japan) placed 10 cm under the water surface and connected to a computer that registered the numbers of light interruptions occurred every 10 min, as described elsewhere (Vera et al. 2014). After this period, fish were sampled at ZT 0.5, 4, 7.5, 12, 16, 20, and 24.5 h (Suppl. Figure 2). At each sampling point, one tank (*n* = 7 fish) from both groups (ML feeding/MD feeding) was sampled. Fish were anaesthetized with clove oil essence (Guinama, Valencia, Spain) at a concentration of 50 µL/L, which was previously diluted in 9 parts of ethanol to improve the dissolution in water. After anesthesia, fish were sacrificed by decapitation to collect samples of hypothalamus and liver, which were frozen in dry ice and subsequently stored at − 80 °C until analyzed. To avoid light contamination during the dark phase, all the sampling procedures at these time points (ZT 12, 16, and 20 h) were performed under a red dim light (*λ* > 600 nm) (de Alba et al. 2019). The sampling was performed in December 2020 and the photoperiod inside the Aquaculture Lab at the sampling time was 10:14 LD (light:dark) and the average water temperature was 19.47 ± 0.17 °C.

### RNA extraction, cDNA synthesis, and real-time quantitative PCR (RT-qPCR) analysis

The hypothalamus and liver were homogenized in TRIzol reagent (Invitrogen, Thermo Fisher Scientific, Waltham, USA) before being mixed with BCP (1-Bromo-3-chloropropane, 99%, Acros Organics, Thermo Fisher Scientific) and centrifuged to obtain a supernatant containing RNA. RNA was then extracted by centrifugation with the addition of isopropanol (Fisher BioReagents, Thermo Fisher Scientific). The RNA was washed twice with 75% ethanol and diluted with DEPC water (Invitrogen, CA, USA). RNA concentration and purity were assessed using spectrometry (Nanodrop® ND 1000, Thermo Fisher Scientific). Then, after the spectrometry, 1U of DNase I (Thermo Fisher) per 1 µg of RNA was added to each sample and incubated at 65ºC for 10 min. Subsequently, cDNA was synthesized using a commercial Reverse Transcriptase kit (QSCRIPT cDNA Synthesis Kit, Quantabio, Beverly, USA) and a thermocycler (MiniAmp Thermal Cycler, Thermo Fisher). The cDNA was utilized for quantitative PCR analysis using Perfecta SYBR Green Fastmin (Quantabio) for the master mix and a real-time thermocycler (7500 RT-PCR system, Applied Biosystem, Foster City, USA) that performed the following steps: 15 min at 95 °C, followed by 40 cycles between 95 °C (15 s) and 60 °C (1 min). Melting curves were run at the end of the qPCR reaction to assess the specificity of the amplicon. All samples were run in duplicate, with each reaction having a final volume of 20 µl. The *Primer 3 Plus software* was used to design *forward* and *reverse* primers (Untergasser et al. 2012) (Table 1). In this study, we analyzed clock genes from both the positive and negative loops (*clock1b*, *bmal1a*, *per1b*, *per2*, *cry1a*, and *cry2*) in both the hypothalamus and liver, and genes involved in epigenetic mechanisms like DNA methylation (*dnmt1* and *dnmt3a*), demethylation (*tet2*, *gadd45aa*, and *mbd4*) and deacetylation (*sirt1*) in the liver samples. All primers were used at a final concentration of 500 nM except *sirt1*, which was added at a final concentration of 200 nM. *β-Actin* and *ef1a* were selected as housekeeping genes. The 2^−ΔΔCt^ method was employed for the analysis of the mRNA expression using the geometric means of the reference genes for the first normalization, while for the second normalization, we used the sample with the lowest Delta-Ct value (Livak and Schmittgen 2001).

**Table 1:** Genes analyzed and primer sequences used for the quantitative PCR analyses

Gene name	Fw	Rv	Acc number
*ef1α*	AGTGAAGCAGCTCATCGTTG	TTGGTGATTTCTCTGAAGCG	AJ866727
*bact*	TCATCACCATCGGCAATGAG	AAGCGTCGCACTTCATGATGC	AY148350
*clock1b*	CCACAGAGCTCCACCCATTA	AAAATCCACTGCTGCCCTTTG	ENSDLA G00005012393
*bmal1a*	TGACGCTAAAACTGGCCTTC	TGCAGAAAAACGACCGTCTG	ENSDLA G00005026433
*per1b*	CATGGTGAAGACGGAAACGGAC	CTTTGGGTGGTTTTCGTCAGG	ENSDLA G00005015065
*per2*	AGCTCCAATGCCTTCAGTCT	ACACATCGGCAGGCATATTT	ENSDLA G00005020824
*cry1a*	AGACCCAGGAGACAAGTTTG	AAGGCCTTCCTCTCCAAATGC	ENSDLA G00005023868
*cry2*	AGCGCCTTCAGACCATTTG	TGCGACATTTGTCCATCTGC	ENSDLA G00005019570
*dnmt1*	ATCAAGCTTGCAGGTGTCAC	TTTGTTGGGTGACGAATGGC	ENSDLA G00005018520
*dnmt3a*	TCATGTGGGA AAACCACAAC	TCTTGTGATGGCTGCATGTGC	ENSDLA G00005018806
*tet2*	TGC CAAC AAGAATGCCATGC	AGTGCC CAGCTTTTGACTTGG	ENSDLA G00005008342
*gadd45aa*	AATTCCAAAAGGCGTGCCTGAC	TGACAGAGGCAACTCCAAAC	ENSDLA G00005028099
*mbd4*	AGGGCCCAAAACACTGTTCCG	TGCAACTCAATGGGGTAACG	ENSDLA G00005035111
*sirt1*	ACGCA AAG TCCCAATGTCAC	ACACTGGGCATT TGGACAAG	ENSDLA G00005013704

### S-Adenosylmethionine (SAM) and S-adenosylhomocysteine (SAH)

Tissue samples (0.088 ± 0.002 g) were first homogenized in cold PBS and centrifuged at 10,000 × g for 15 min at 4 °C, and the resulting supernatants were stored at − 80 °C until analyzed. Levels of S-adenosylmethionine (SAM) and S-adenosylhomocysteine (SAH) in the liver samples were measured using a commercial ELISA kit (Cell Biolabs Inc., San Diego CA, USA, ref number MET-5151-C), following the manufacturer’s instructions.

### Data analysis

To assess statistically significant differences between time points (ZT) and groups (ML and MD), all the biological variables studied underwent two-way ANOVA analysis followed by a Tukey post hoc test using SPSS software (v. 28.0.1.1, IBM, Armonk, USA). Additionally, data obtained from SAM and SAH analysis were pooled separately for light and dark phases to compare ML and MD groups during the same phase using a Student’s *t*-test. Daily patterns of locomotor activity (percentage of diurnalism) data were subjected to a Student’s *t*-test to evaluate statistically significant differences between ML and MD tanks. The significance threshold was set at α = 0.05 for all tests, and results are expressed as mean ± SEM. The presence of a daily rhythm was assessed using Cosinor analysis with El Temps software (v. 313, Prof. Díez-Noguera, University of Barcelona, Spain). The Cosinor analysis involves using a least square method to approximate time series data with a cosine function. The model is expressed as *Y* = *M* + *A* × *(COS (Ωt* + φ)). In the formula, M represents the mesor, *A* represents the amplitude, *Ω* represents the angular frequency (with 360°/24 h for daily rhythms), and φ represents the acrophases. The Cosinor analysis also defines the statistical significance of the rhythm since an *F* test of the variance is described for the waveform versus a straight line of zero amplitude. Therefore, if *p* < 0.05, then the null hypothesis is rejected determining a statistically significant rhythm of the given time series data (Refinetti et al. 2007; Portaluppi et al. 2008).

El Temps software was also employed to analyze locomotor activity recorded during the experiment and to plot actograms and waveforms. Figures were generated using GraphPad.

## Results

### Daily patterns of locomotor activity

**Fig. 1:**
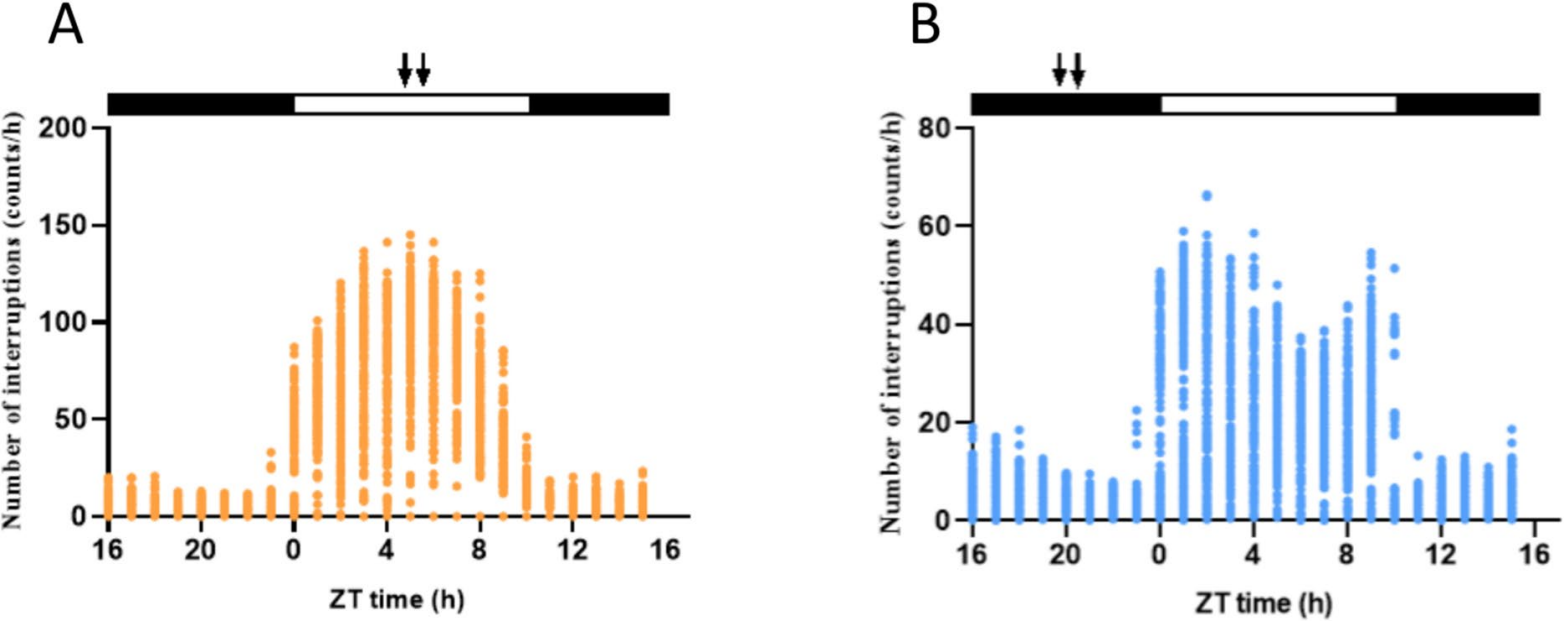
Average diel profile of the locomotor activity of two groups of European sea bass maintained in a 10:14 LD cycle and fed during the middle of the light (ML) (**A**) or dark (MD) phase (**B**). Each point in the mean waveform has been calculated as the mean ± SD from 10-min binned data across all the experimental days (*n* = 30) and tanks (*n* = 7). The white and black bars above represent the light and dark phases, respectively, while the arrows represent the feeding times (ML and MD)

All European sea bass groups exhibited a predominantly diurnal activity pattern regardless of feeding time. However, fish fed at ML showed 72.59 ± 1.57% of their daily activity during the light phase, whereas fish from the MD group displayed a lower percentage of total activity during the day, 55.33 ± 6.75% (Fig. 1). This difference was statistically significant when comparing the activity of ML and MD groups during the light phase (% of diurnalism) (*p* < 0.0001) (*t*-test *p* < 0.05). Additionally, the shape of the daily average of activity differed, as it was centered around feeding time in the ML group, while the MD group exhibited two peaks during the light phase, both close to lighting transitions (Fig. 1).

### Hypothalamic molecular clock

**Fig. 2:**
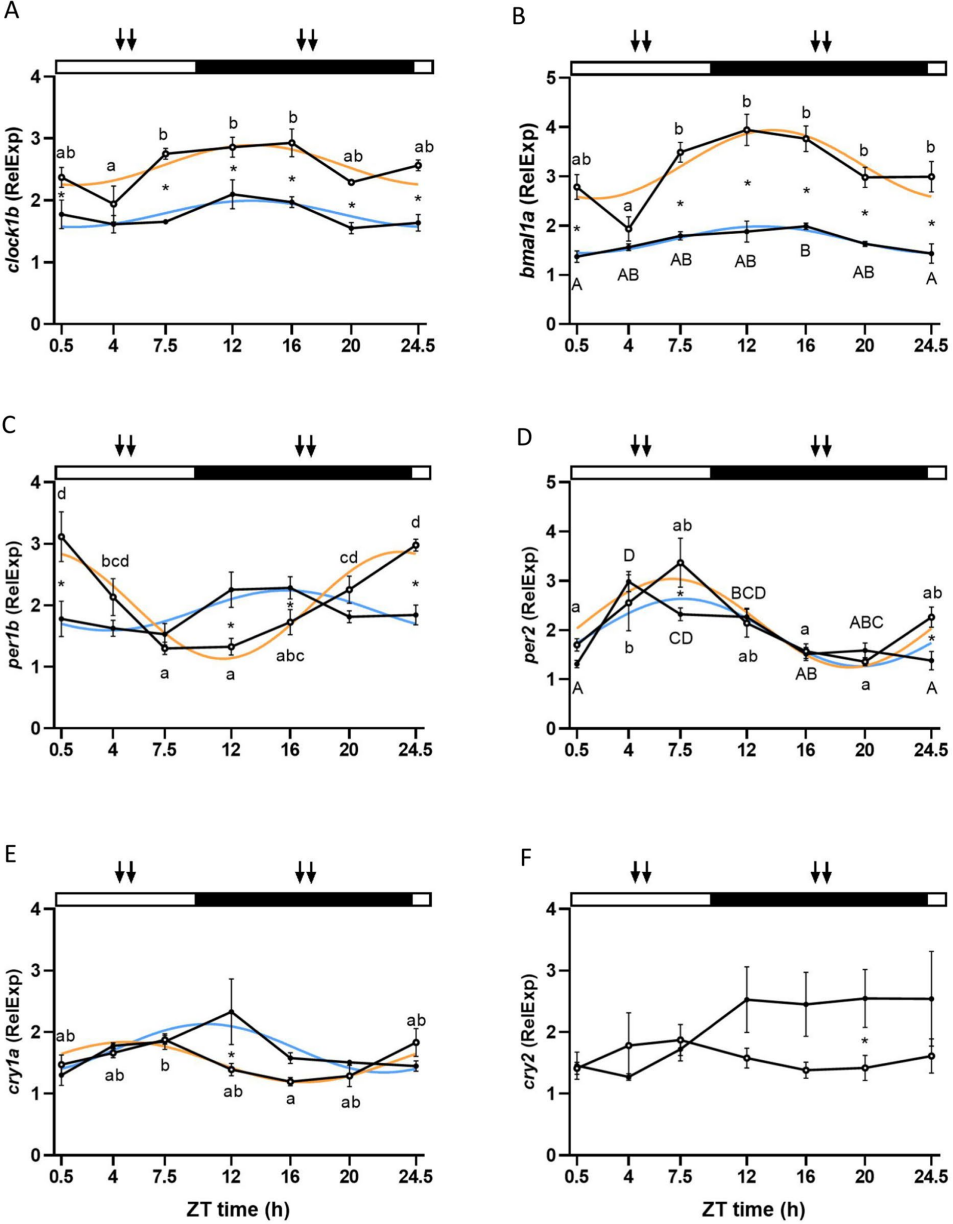
Daily variations in the relative mRNA expression (fold change) of *clock1b* (**A**), *bmal1a* (**B**), *per1b* (**C**), *per2* (**D**), *cry1a* (**E**), and *cry2* (**F**) in the hypothalamus of two groups of European sea bass maintained in a 10:14 LD cycle and fed during the middle of the light (ML) or dark (MD) phase. White circles (○) represent the ML group, while the MD group is represented with black dots (●). The adjustment to a sinusoidal rhythm (Cosinor, *p* < 0.05), when significant, is represented by orange and light blue lines for ML and MD groups, respectively. Statistically significant differences between ZT points within the ML and MD groups are represented by different lower- and upper-case letters (two-way ANOVA), respectively. The asterisks indicate significant differences between ML and MD groups at the same time point (two-way ANOVA). The white and black bars above the graphs represent the light and dark phases, respectively, while the arrows represent the feeding time for each group. The x-axis represents the time scale as ZT (*zeitgeber* time, h). All data are represented as mean ± SEM (*n* = 7 fish per point)

Clock genes from the positive (*clock1b*, *bmal1a*) and negative loops (*per1b*, *per2*, *cry1a*, and *cry2*) of the molecular clock were analyzed in the hypothalamus of sea bass from the two groups. Regarding the genes from the positive loop, *clock1b* and *bmal1a* exhibited a similar pattern. Both genes displayed daily rhythms in both ML and MD groups (Cosinor, *p* < 0.05) with similar nocturnal acrophases, located around 3 h after light offset (ML: *clock1b* ZT 13:21 h, *bmal1a* ZT 13:46 h; MD: *clock1b* ZT 13:18 h, *bmal1a* ZT 13:06 h) (Fig. 2a and b; Table 2). Significant differences throughout the 24-h cycle between time points were observed for both groups except for *clock1b* in MD feeding (two-way ANOVA, *p* < 0.05) (Fig. 2a and b). Moreover, feeding influenced *clock1b* and *bmal1a* similarly, with higher expression levels in the ML feeding group compared to MD (two-way ANOVA *p* < 0.05) (Fig. 2a and b) (Suppl. Table 1).

**Table 2:** Resume of the Cosinor analysis results of the biological variables analyzed in the present study. Data are only indicated for significant rhythms (Cosinor, *p* < 0.05): *p* value, mesor, amplitude, and acrophase (indicated in ZT time). Data are expressed as value ± fiducial limits (set at 95%)

Tissue and histological factor	Experimental group	*p* values	Mesor	Amplitude	Acrophase (ZT hours)
Hypothalamus					
*clock*1b	ML	0.0072	2.57 ± 0.15	0.32 ± 0.24	13:21 ± 3:33
	MD	0.03208	1.78 ± 0.11	0.21 ± 0.19	13:18 ± 4:31
*bmal1*a	ML	0.00024	3.24 ± 0.23	0.69 ± 0.37	13:46 ± 2:33
	MD	0.00109	1.71 ± 0.10	0.27 ± 0.17	13:06 ± 2:40
*per1b*	ML	0.000	2.01 ± 0.18	0.87 ± 0.32	23:34 ± 1:24
	MD	0.01396	1.92 ± 0.15	0.32 ± 0.26	15:51 ± 2:07
*per2*	ML	0.00005	2.13 ± 0.23	0.90 ± 0.44	7:08 ± 1:42
	MD	0.00007	1.94 ± 0.18	0.68 ± 0.35	4:46 ± 1:30
*cry1a*	ML	0.00445	1.51 ± 0.12	0.32 ± 0.23	5:05 ± 2:23
	MD	0.01844	1.73 ± 0.18	0.39 ± 0.23	10:23 ± 3:30
*cry2*	ML	0.19821			
	MD	0.11578			
Liver					
*clock1b*	ML	0.00219	4.95 ± 1.45	3.49 ± 2.49	12:49 ± 3:01
	MD	0.12196			
*bmal1a*	ML	0.00412	3.47 ± 0.72	1.8 ± 1.21	14:58 ± 3:00
	MD	0.15100			
*per1b*	ML	0.02025	6.29 ± 1.07	2.27 ± 1.96	18:43 ± 4:07
	MD	0.65381			
*per2*	ML	0.02067	6.16 ± 1.09	2.21 ± 1.92	23:17 ± 3:22
	MD	0.00628	9.29 ± 2.45	5.68 ± 4.25	5:09 ± 3:32
*cry1a*	ML	0.03223	6.68 ± 0.8	1.57 ± 1.46	9:50 ± 3:09
	MD	0.34997			
*cry2*	ML	0.02714	2.61 ± 0.48	0.93 ± 0.84	11:55 ± 3:01
	MD	0.83407			
*dnmt1*	ML	0.01397	3.61 ± 0.61	1.25 ± 1.08	15:10 ± 4:06
	MD	0.08701			
d*nmt3*a	ML	0.02922	6.33 ± 1.62	3.02 ± 2.76	15:29 ± 4:30
	MD	0.04385	11.28 ± 3.18	5.57 ± 5.44	17:29 ± 4
*tet2*	ML	0.00178	3.72 ± 0.85	2.27 ± 1.5	16:37 ± 3:01
	MD	0.34229			
*gadd45aa*	ML	0.00005	3.65 ± 0.63	2.18 ± 1.1	16:10 ± 2:10
	MD	0.11877			
*mbd4*	ML	0.00001	4.49 ± 0.84	3.40 ± 1.47	16:25 ± 1:48
	MD	0.33270			
*sirt1*	ML	0.03910	6.06 ± 2.69	4.84 ± 4.62	12:05 ± 4:32
	MD	0.07929			
SAM	ML	0.31358			
	MD	0.55693			
SAH	ML	0.51030			
	MD	0.23000			
SAM/SAH	ML	0.36302			
	MD	0.54500			

As for the negative loop*, per1b* and *per2* exhibited daily rhythms in both ML and MD feeding groups (Cosinor, *p* < 0.05) but with different acrophases. In both groups, *per1b* peaked during the dark phase (ZT 23:34 h and 15:51 h for ML and MD, respectively) while *per2* peaked during the light phase (ZT 7:08 h and 4:46 h for ML and MD, respectively). In both cases, an advance of the acrophases in the MD group compared to the ML group was evident (Fig. 2c and d, Table 2). This was mainly evident for *per1b*, which displayed a shift of around 7 h between ML and MD feeding groups (Fig. 2c). Like the positive loop, both genes showed significant differences between time points throughout the 24-h cycle (two-way ANOVA, *p* < 0.05) (Fig. 2c and d). Additionally, a statistically significant interaction between feeding time and sampling points was observed for both genes (two-way ANOVA *p* < 0.05) (Suppl. Table 1).

The other two components of the negative loop of the hypothalamic circadian clock exhibited a different pattern*. Cry1a* showed rhythms both in ML and MD groups (Cosinor, *p* < 0.05), with partially shifted acrophases since in the ML group, it peaked during the light phase, at ZT 5:05 h, while in the MD group, *cry1a* expression peaked at the beginning of the dark phase (ZT 10:23 h) (Fig. 2e and f; Table 2). Conversely, *cry2* did not exhibit any significant rhythm (Cosinor, *p* > 0.05). Moreover, significant differences between sampling points were observed only for *cry1a* in the ML group (two-way ANOVA, *p* < 0.05). Significant differences between ML and MD groups were also found at ZT 12 h for *cry1a* and ZT 20 h for *cry2* (Fig. 2e and f). Finally, a significant interaction between feeding and sampling times was observed for *cry1a* mRNA expression (two-way ANOVA, *p* < 0.05) (Suppl. Table 1).

### Liver molecular clock

**Fig. 3:**
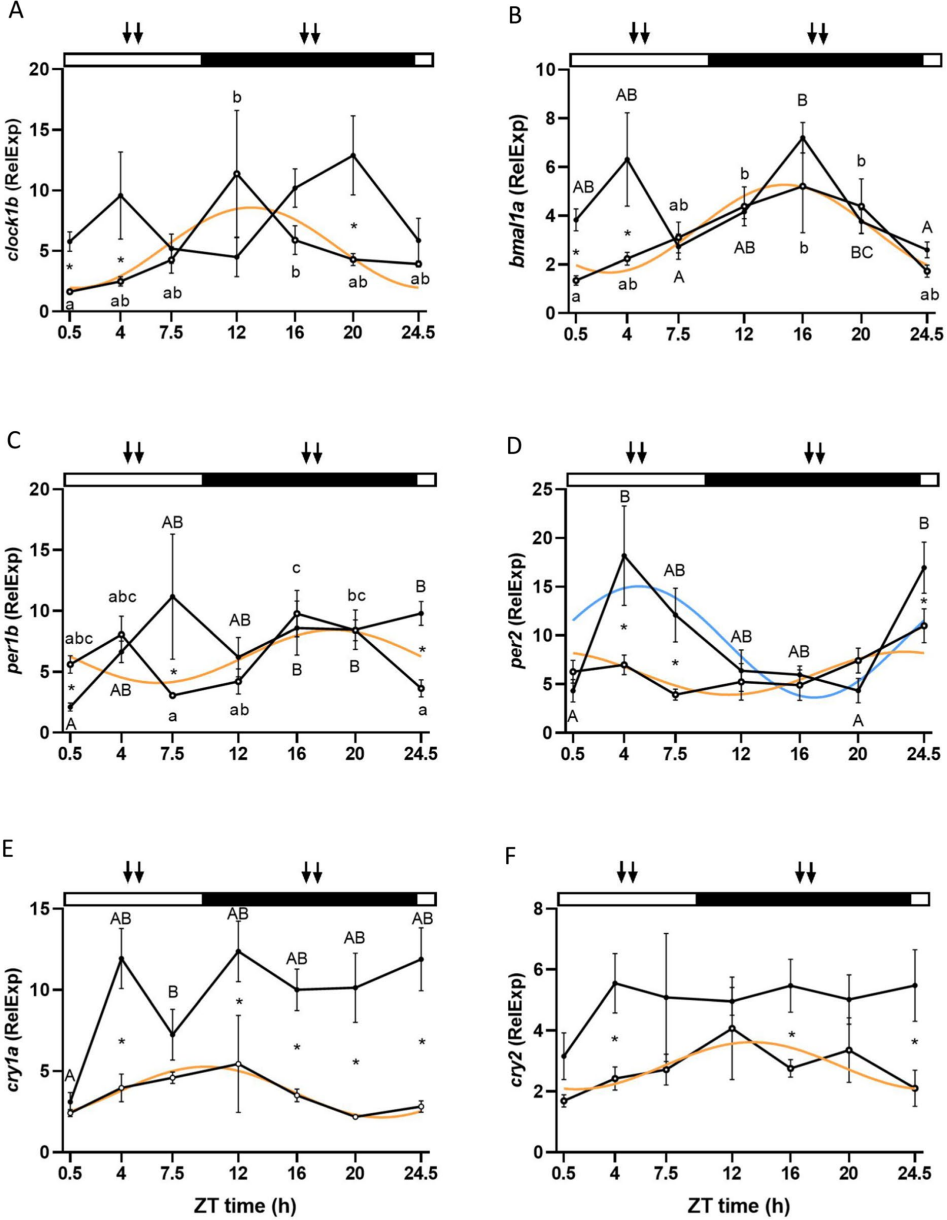
Daily variations in the relative mRNA expression (fold change) of *clock1b* (**A**), *bmal1a* (**B**), *per1b* (**C**), *per2* (**D**), *cry1a* (**E**), and *cry2* (**F**) in the liver of two groups of European sea bass maintained in a 10:14 LD cycle and fed during the middle of the light (ML) or dark (MD) phase. White circles (○) represent the ML group, while the MD group is represented with black dots (●). The adjustment to a sinusoidal rhythm (Cosinor, *p* < 0.05), when significant, is represented by orange and light blue lines for ML and MD groups, respectively. Statistically significant differences between ZT points within the ML and MD groups are represented by different lower- and upper-case letters (two-way ANOVA), respectively. The asterisks indicate significant differences between ML and MD groups at the same time point (two-way ANOVA). The white and black bars above the graphs represent the light and dark phases, respectively, while the arrows represent the feeding time for each group. The x-axis represents the time scale as ZT (*zeitgeber* time, h). All data are represented as mean ± SEM (*n* = 7 fish per point)

Regarding the molecular clock in the liver, the genes from the positive loop, *clock1b* and *bmal1a*, presented different patterns depending on the feeding regime used. Both genes presented a significant rhythm in the ML group (Cosinor, *p* < 0.05) with nocturnal acrophases located at ZT 12:49 h for *clock1b* and ZT 14:58 h for *bmal1a* (Fig. 3a and b, Table 2). In contrast*,* no significant rhythms were observed in the MD group (Cosinor, *p* > 0.05). However, significant differences between time points were found for *clock1b* and *bmal1a* in the ML group and *bmal1a* in the MD group (two-way ANOVA, *p* < 0.05). Concerning the differences between ML and MD, statistically significant differences were found for *clock1b* at ZT 0.5, 7.5, and 20 h, while differences in *bmal1a* expression were found only at the beginning of the day (ZT 0.5 and 4 h) (two-way ANOVA, *p* < 0.05) (Fig. 3a and b). Feeding time and sampling points significantly affected both genes, but not the interaction between them (two-way ANOVA *p* < 0.05) (Suppl. Table 1).

Regarding the negative loop of the liver molecular clock, *per1b* and *per2* mRNA expression presented rhythms in the ML group (Cosinor, *p* < 0.05), with nocturnal acrophases located at ZT 18:43 h and 23:17 h, respectively. However, only *per2* displayed rhythmicity in the MD group, showing a diurnal acrophase located at ZT 5:09 h (Fig. 3c and d, Table 2). The two-way ANOVA analysis revealed significant differences between sampling times for *per1b* (ML and MD groups) and *per2* (only MD). Furthermore, significant differences between ML and MD groups on the mRNA expression of these genes were found at some ZT points: 0.5, 7.5, and 20 h for *per1b*, and 4, 7.5, and 24.5 h for *per2* (Fig. 3c and d) (two-way ANOVA *p* < 0.05) (Suppl. Table 1).

Finally, *cry1a* and *cry2* presented a similar trend as observed for the other clock genes in the liver since a significant rhythmicity was only found in the ML group (Cosinor, *p* < 0.05). In this group, the acrophases were located at the transition between light and dark phases for *cry1a* (ZT 9:50 h) and at the beginning of the dark phase for *cry2* (ZT 11:55 h) (Fig. 3e and f, Table 2). Additionally, significant differences between sampling points were found only for *cry1a* at the MD group, but the differences between the two feeding conditions (ML vs. MD) were observed at several sampling points for both genes: ZT 4, 12, 16, 20, and 24.5 h for *cry1a*, and ZT 4, 16, and 24.5 h for *cry2* (two-way ANOVA, *p* < 0.05) (Fig. 3e and f).

### Genes involved in methylation (*dnmt1*, *dnmt3a*), demethylation (*tet2*, *gadd45aa*, *mbd4*), and deacetylation processes (*sirt1*) in the liver

**Fig. 4:**
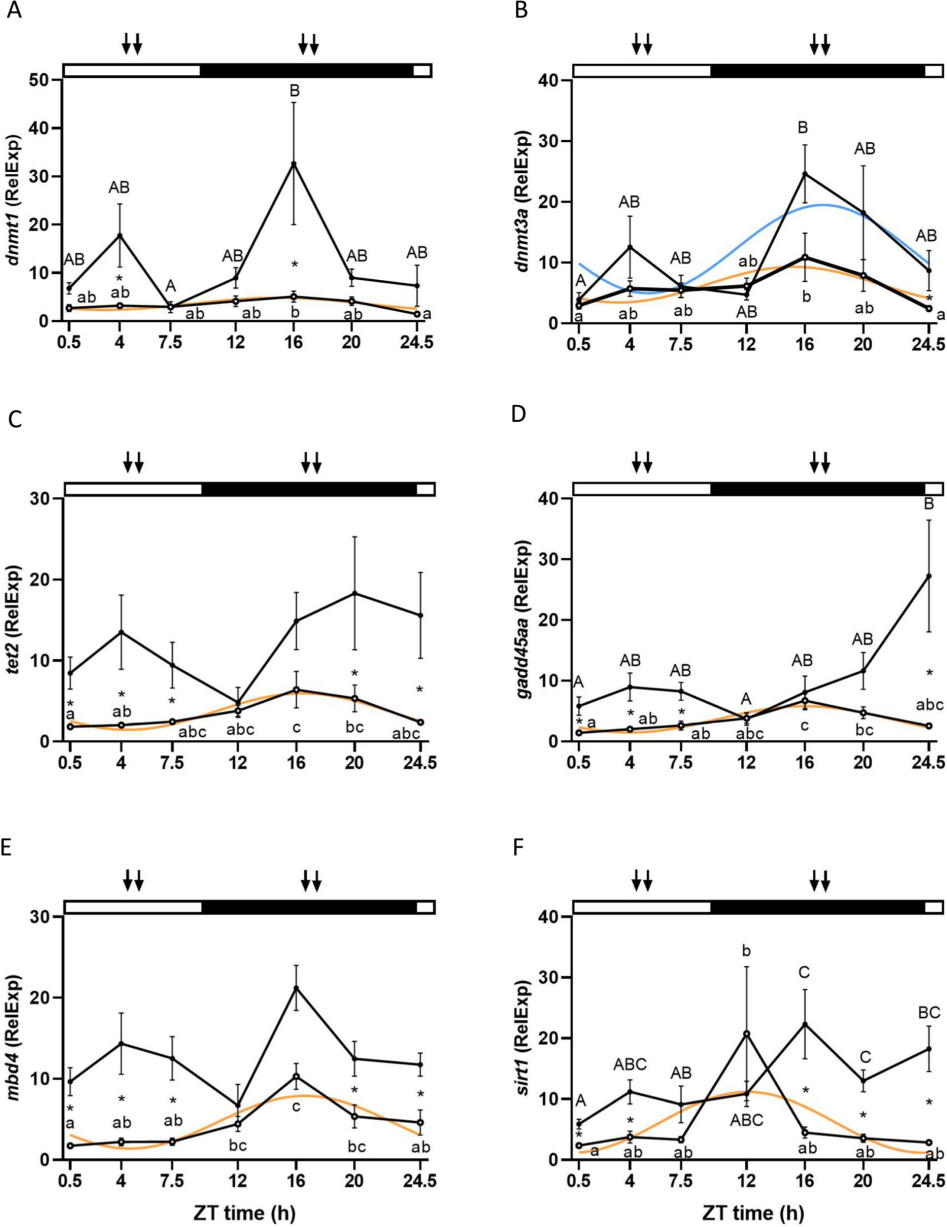
Daily variations in the relative mRNA expression (fold change) of *dnmt1* (**A**), *dnmt3a* (**B**), *tet2* (**C**), *gadd45aa* (**D**), *mbd4* (**E**), and *sirt1* (**F**) in the liver of two groups of European sea bass maintained in a 10:14 LD cycle and fed during the middle of the light (ML) or dark (MD) phase. White circles (○) represent the ML group, while the MD group is represented with black dots (●). The adjustment to a sinusoidal rhythm (Cosinor, *p* < 0.05), when significant, is represented by orange and light blue lines for ML and MD groups, respectively. Statistically significant differences between ZT points within the ML and MD groups are represented by different lower- and upper-case letters (two-way ANOVA), respectively. The asterisks indicate significant differences between ML and MD groups at the same time point (two-way ANOVA). The white and black bars above the graphs represent the light and dark phases, respectively, while the arrows represent the feeding time for each group. The x-axis represents the time scale as ZT (*zeitgeber* time, h). All data are represented as mean ± SEM (*n* = 7 fish per point)

Genes involved in methylation and demethylation processes were analyzed in the liver. Concerning methylation, *dnmt1* and *dnmt3a* displayed daily rhythms with some distinction. *Dnmt1* displayed a significant rhythm solely in the ML group (Cosinor, *p* < 0.05) whereas *dnmt3a* exhibited a rhythm in both ML and MD groups (Cosinor, *p* < 0.05). The acrophases for both genes were located during the first half of the dark phase. In the ML group, *dnmt1* and *dnmt3a* presented very close acrophases, which were located at ZT 15:24 h and ZT 15:10 h, respectively. The acrophase of *dnmt3a* in the MD group was located at ZT 17:29 h, slightly delayed when compared to ML group (Fig. 4a and b, Table 2). Both genes displayed significant differences between sampling points (two-way ANOVA, *p* < 0.05) and the differences between ML and MD group at the same time point were significant at ZT 4 and 16 h for *dnmt1* and ZT 24.5 h for *dnmt3a* (Fig. 4a and b). Feeding and sampling time had a significant effect on methylation genes, but not the interaction between them (two-way ANOVA, *p* < 0.05) (Suppl. Table 1).

Regarding the daily rhythms in demethylation processes, all genes analyzed (*tet2, gadd45aa*, and *mbd4*) displayed a similar pattern. The rhythm was only present in the ML group for all three genes, with nocturnal acrophases occurring closely together and situated towards the middle of the dark phase. Specifically, *tet2* peaked at ZT 16:37 h, *gadd45aa* at ZT 16:10 h and *mbd4* at ZT 16:25 h (Cosinor *p* < 0.05) (Fig. 4c–e, Table 2). In addition, all genes presented significant differences between time points throughout the 24 h in the ML group, but only *gadd45aa* conserved this pattern also in the MD group (two-way ANOVA, *p* < 0.05) (Fig. 4c–e). All genes presented significant differences between ML and MD groups at ZT 0.5, 4, 7.5, 20, and 24.5 h, with the exception of *gadd45aa* at ZT 20 h (two-way ANOVA, *p* < 0.05) (Fig. 4c–e). Feeding time significantly influenced the three genes, while only *gadd45aa* was also affected by the interaction between feeding and sampling times (two-way ANOVA, *p* < 0.05) (Suppl. Table 1).

In addition to methylation and demethylation, the mRNA expression of *sirt1*, a gene involved in acetylation processes, was examined in the liver. This gene presented a significant daily rhythm solely in the ML group (Cosinor, *p* < 0.05), with its acrophase occurring at the beginning of the dark phase (ZT 12:05 h) (Fig. 4F, Table 2). Significant differences between time points throughout the 24 h were observed in both feeding regimes. Furthermore, significant differences in *sirt1* expression between the ML and MD groups were found at ZT 0.5, 4, 16, 20, and 24.5 h (Fig. 4f) (two-way ANOVA, *p* < 0.05). The mRNA expression of *sirt1* was significantly influenced by feeding and sampling times, but not by their interaction (two-way ANOVA, *p* < 0.05) (Suppl. Table 1).

### S-Adenosyl methionine (SAM), S-adenosyl homocysteine (SAH), and methylation potential (SAM/SAH) in the liver

**Fig. 5:**
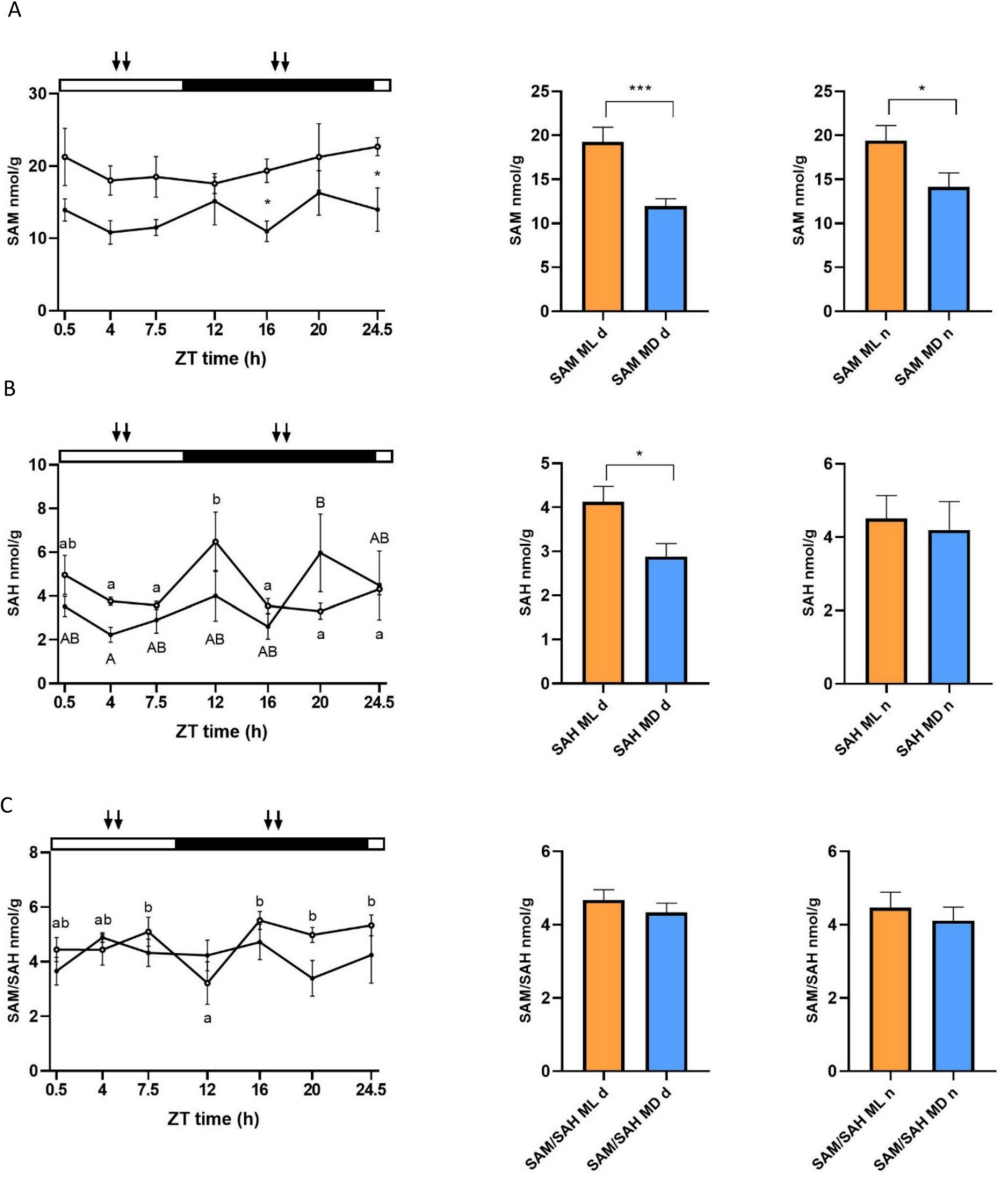
Daily variations of S-adenosyl methionine (SAM) (**A**), S-adenosyl homocysteine (SAH) (**B**), and the methylation potential represented by the SAM/SAH ratio (**C**) in the liver of two groups of European sea bass maintained in a 10:14 LD cycle and fed during the middle of the light (ML) or dark (MD) phase. White circles (○) represent the ML group, while the MD group is represented with black dots (●). Statistically significant differences between ZT points within the ML and MD groups are represented by different lower- and upper-case letters (two-way ANOVA), respectively. The asterisks indicate significant differences between ML and MD groups at the same time point (two-way ANOVA). The white and black bars above the graphs represent the light and dark phases, respectively, while the arrows represent the feeding time for each group. The x-axis represents the time scale as ZT (*zeitgeber* time, h). In addition, to the right, day-night differences in SAM (**A**), SAH (**B**), and the SAM/SAH ratio (**C**) have been represented. Data from ML and MD groups are represented by white and dark bars, respectively. Values were obtained by pooling all data from the light (*d*) or the dark phase (*n*) and were compared by means of Student’s *t*-test. Statistically significant differences between groups (*p* < 0.05) are indicated with an asterisk. All data are represented as mean ± SEM (*n* = 7 fish per point)

Besides gene expression, SAM and SAH abundance, as well as their ratio (SAM/SAH), were analyzed in the liver. None of these nutrient related factors presented any significant rhythmicity (Cosinor *p* > 0.05) (Fig. 5, Table 2). SAH (ML and MD groups) and the SAM/SAH ratio (in the ML group) presented significant differences between time points (two-way ANOVA, *p* < 0.05) (Fig. 5, Table 2). In addition, significant differences between ML and MD groups were observed for SAM at ZT 16 and 24.5 h (Fig. 5), which was also affected by the feeding regime, with fish from the ML group showing higher SAM levels than MD (two-way ANOVA *p* < 0.05) (Suppl. Table 2). This difference could also be observed by pooling the data from the day (*d*) or night (*n*) phases (Fig. 5, right). SAM levels were higher in the ML group than MD at both day and night (*t*-test, *p* < 0.05) (Fig. 5A), while SAH levels were higher in the ML group than MD only during the light phase (*t*-test, *p* < 0.05) (Fig. 5B). On the contrary, the SAM/SAH ratio presented no differences either during the day or at night (*t*-test, *p* > 0.05) (Fig. 5C).

## Discussion

**Fig. 6:**
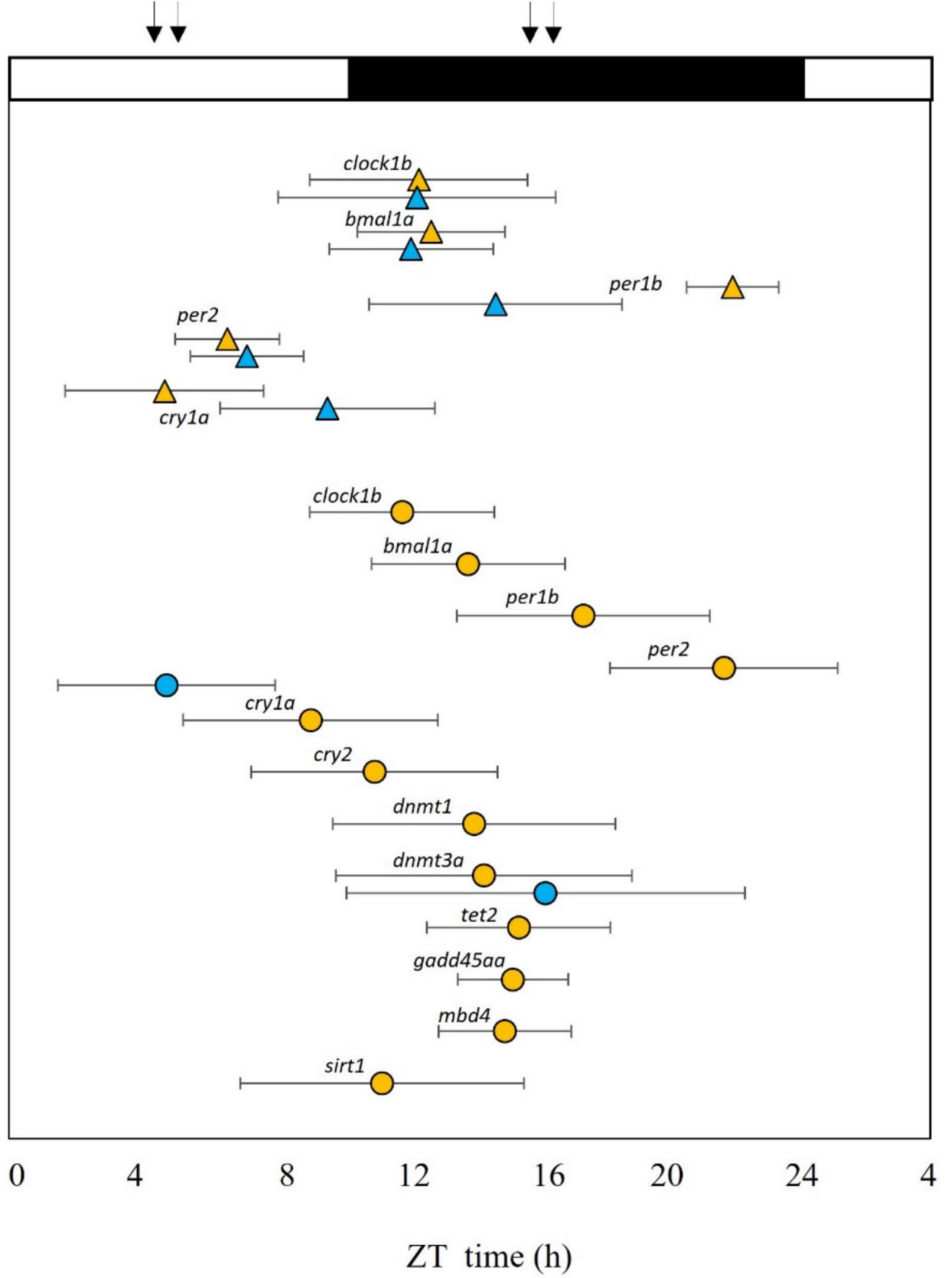
Map of acrophases of the genes analyzed in the present study and involved in the molecular clock, methylation*,* demethylation, acetylation, and methylation potential. The acrophase is indicated only for the statistically significant rhythms (Cosinor *p* < 0.05) and the name of each gene is indicated near the correspondent marker. ML group parameters are represented with orange triangles (hypothalamus) or circles (liver), while MD group parameters are represented with blue triangles (hypothalamus) or circles (liver). The x-axis represents the time scale, which is expressed as ZT (*zeitgeber*) time and where ZT 0 corresponds to the light onset. The white and dark bars above the figure represent the light and dark phases of the LD cycle (10:14 LD), respectively, and the black arrows represent the feeding times

In the present research we examined the effect of feeding time on positive (*clock1b*, *bmal1a*) and negative (*per1b*, *per2*, *cry1a*, *cry2*) loops of the molecular clock in the liver of European sea bass, along with the effect on behavior. Additionally, we demonstrated the existence of daily rhythms in the expression of various genes involved in epigenetic processes, such as methylation (*dnmt1*, *dnmt3a*), demethylation (*tet2*, *gadd45aa*, *mbd4*), and deacetylation (*sirt1*) in the liver of the sea bass (Fig. 6). Feeding time had also a strong influence on the rhythms of these genes, revealing a possible interplay between the circadian system and the epigenetic processes. In this research, we also focused on the central pacemaker (hypothalamus) describing the rhythm of the clock genes. Our analysis revealed that feeding significantly influences the clock in peripheral tissues but has a lesser impact on the brain of the species studied, the European sea bass (Fig. 6).

In their natural environment, some fish species can exhibit dual behavior during seasonal alternation, among them the European sea bass (Sánchez-Vázquez et al. 1998). One of the most effective methods to induce such behavioral shifts under laboratory conditions is through the manipulation of feeding time (Del Pozo et al. 2012). In our study, a significant reduction in the activity during the light phase was observed in fish from the MD group, although it was not enough to be considered a clear shift in activity patterns (from diurnal to nocturnal). Factors other than food may have influenced these results. For example, in the natural environment, gradual changes in photoperiod are typically accompanied by changes in temperature, and both factors contribute to shift the activity patterns of European sea bass (Sánchez-Vázquez et al. 1998). In our experiment, however, the recorded temperature was not as low as expected for December and this could have influenced the behavior observed.

Recently, there has been growing interest in the relationship between the circadian system and the epigenetic mechanisms of DNA methylation. These mechanisms are implicated in the transcriptional regulation of clock genes at various levels. Previous studies have suggested that Dnmt3a may play a role in the methylation of the promoter of *Bmal1* in certain diseases, leading to its silencing (Satou et al. 2013). Additionally, DNA methyltransferases are intertwined with cellular metabolism, as they depend on the availability of methyl groups, thus linking this epigenetic mechanism to feeding behavior. In our investigation, we aimed to determine whether genes involved in the clock and epigenetic mechanisms of the sea bass exhibit daily rhythms and how feeding time may influence these systems.

The clock genes work at the molecular level creating a self-sustainable molecular clock based on positive and negative feedback loops (Vatine et al. 2011). Food can be a powerful synchronizer for the molecular clock of peripheral oscillators both in mammals and fish (López-Olmeda 2017). In the liver, feeding time significantly influenced the molecular clock, as all the analyzed genes from both the positive and negative loops exhibited rhythms in the ML group, while only *per2* maintained the rhythm in the MD group. Notably, *per2* has been previously characterized as light-dependent in zebrafish (Pando and Sassone-Corsi 2002; Vatine et al. 2011). However, in the present study, we observed a 6-h shift in its acrophase, suggesting an effect of feeding time for this gene in the liver of European sea bass, as reported for other species such as gilthead sea bream (Vera et al. 2013). Interestingly, nocturnal feeding in diurnal sea bass suppressed the rhythmicity of certain clock genes in the liver, analogous to observations in some clock genes in other species such as Nile tilapia (*bmal1a* and *per1b*) and gilthead seabream (*per2*) (Vera et al. 2013; Costa et al. 2016). The complexity of the circadian system suggests that additional variables such as seasonality might be involved, as evidenced in previous studies on the European sea bass pituitary (Herrero and Lepesant 2014). Furthermore, we examined the daily rhythms of locomotor activity as an indicator of the output signal from the circadian system in sea bass. Previous studies have demonstrated that periodic feeding alone serves as an important *zeitgeber* for the locomotor activity rhythms of fish (López-Olmeda 2017). When conflicting with the LD cycle, nocturnal feeding may lead to a shift from diurnal to nocturnal activity, as observed in certain fish species, such as the gilthead seabream (Montoya et al. 2010). However, in some species, the alteration in behavioral patterns is not consistently complete. For example, goldfish predominantly exhibited diurnal locomotor activity regardless of feeding time, although a significant reduction in diurnalism was noted in fish fed during the middle of the night compared to those fed during the light phase (Gómez-Boronat et al. 2018). This aligns with the findings of the current research on European sea bass behavior, where both groups remained primarily diurnal, but nighttime feeding diminished the percentage of diurnalism and altered the shape of the daily rhythm of activity. Collectively, these results underscore the influence of feeding time on clock genes and its impact on overt rhythms such as the daily patterns of locomotor activity.

To the best of our knowledge, rhythms in the expression of genes involved in DNA methylation and demethylation in fish have only been previously demonstrated in zebrafish gonads (Paredes et al. 2018). In our study, European sea bass fed during the light phase displayed rhythmic expression of genes involved in methylation (*dnmt1*, *dnmt3a*) and demethylation processes (*tet2*, *gadd45aa*, and *mbd4*). In contrast, only *dnmt3a* exhibited rhythms in the liver of fish fed during the dark phase. In all cases, the acrophases were situated during the dark phase, which corresponds to the resting phase of the sea bass used in the experiment, as evidenced by the locomotor activity records. This finding aligns with the results of a previous study in zebrafish ovaries, where genes involved in methylation and demethylation also exhibited daily rhythms with peak values during the dark/resting phase (Paredes et al. 2018). In mouse liver, the peak expression of *dnmt3a* occurs during the light phase, correlating with the variation in DNA methylation levels (Xia et al. 2015). Mice, unlike zebrafish or sea bass, are nocturnal animals and are more active during the night, resting during the light phase (Robinson-Junker et al. 2018). Thus, in both mammals and fish, the acrophase of genes involved in methylation processes appears to be inversely related to their activity phase, suggesting that the epigenetic landscape may undergo more significant remodeling during the animal’s resting phase.

The acrophases of genes involved in methylation and demethylation closely resemble those displayed by *clock1b* and *bmal1a*, supporting the idea of a connection between the two systems in the liver of sea bass, as previously found in mice (Maekawa et al. 2012). Among all genes analyzed in the epigenetic mechanisms, *dnmt3a* was the only gene exhibiting a rhythm in fish fed during the dark phase, thereby escaping the effects elicited by MD feeding that seemed to suppress the rhythms in the other genes analyzed. This suggests that *dnmt3a* rhythms may not respond to food signals and could be regulated by other systemic mechanisms. For instance, in mammals, *Dnmt3a* rhythms seem to be regulated by the daily variations in SAM levels, as well as the SAM/SAH ratio (Zhang et al. 2018). The fact that *dnmt3a* expression was not altered by feeding time and maintained its rhythmicity also suggests the high importance of this gene in circadian mechanisms. In mammals, *dnmt3a* is identified as a potential transcriptional regulator for *bmal1a* (Satou et al. 2013). Moreover, knocking out DNMT3a in a cell line induced differences in the circadian periods of this cell line (Li et al. 2020), supporting the idea of the importance of this protein in the maintenance or stability of the molecular clock. On the other hand, the rhythm of *dnmt1* was not maintained in the MD group, suggesting that, at least in the sea bass liver, the maintenance of methylation driven by *dnmt1* could follow a different regulation than de novo methylation driven by *dnmt3a*.

Acetylation plays an important role in regulating the circadian system through *sirt1*, whose histone deacetylase activity compensates for CLOCK’s acetylase function (Nakahata et al. 2008). In mammals, SIRT1 is described as a NAD + -dependent cytoplasmic enzyme that relies on cellular energy (Suave et al. 2006), suggesting an essential role of feeding in its regulation. Moreover, *Sirt1* gene expression and its protein levels do not display rhythmicity in mice (Nakahata et al. 2008), but NAD + presents a clear oscillation in this species, ultimately regulating the daily activity of SIRT1 (Ramsey et al. 2009; Bellet et al. 2013). In our study, contrary to observations in mammals, *sirt1* mRNA expression exhibited a daily rhythm in the European sea bass. Additionally, daily rhythms in *sirt1* expression were suppressed in fish fed during the MD period, indicating a significant effect of feeding and possibly of the metabolic state through NAD + levels, as observed in mammals. However, further research would be required in the future to test this hypothesis regarding the role of NAD + in fish.

SAM, SAH, and the methylation potential (SAM/SAH ratio) did not display a daily rhythm, but there were some differences observed when comparing the phase of the LD cycle and feeding time. SAM showed differences between ML and MD fed sea bass, suggesting a distinct utilization of energy provided by the diet, as ATP is required for the process of methionine activation to synthesize SAM (Froese et al. 2019). Additionally, SAH exhibited nocturnal peaks, with higher values at night than during the day, at ZT 12 and 20 h for ML and MD groups, respectively. Similar day-night differences have been previously reported in mammals (Xia et al. 2015). These peaks were inversely related to the lowest values in the methylation potential indicated by SAM/SAH in the sea bass liver. The low values in the methylation potential during the dark phase would also suggest a higher methyltransferase activity at this time of day, correlating with the higher *dnmt3*a expression observed during this phase.

Different studies on fish have reported that the central oscillators in the brain do not seem to respond in the same way to feeding time (López-Olmeda 2017). In our study, in the hypothalamus, the rhythm is present in most of the clock genes analyzed (*clock1b*, *bmal1a*, *per1b*, *per2*, and *cry1a*) in both ML and MD groups. In sea bass fed at ML, genes from the positive loop (*clock1b* and *bmal1a*) present an acrophase around the beginning of the dark phase, while the acrophases of genes from the negative loop are shifted and peak around the end of the night (*per1b*) or during the light phase (*per2*, *cry1a*). These rhythms and their phases are conserved among teleost fish and have been reported for several species such as the zebrafish (Vatine et al. 2011), Nile tilapia (Costa et al. 2016), gilthead seabream (Vera et al. 2013), goldfish (Gómez-Boronat et al. 2018), and also previously for the European sea bass (Sanchez et al. 2010, Del Pozo et al. 2012; Herrero and Lepesant 2014). Regarding fish fed at MD, *clock1b* and *bmal1a* presented similar acrophases to the ML group, suggesting that feeding time alone probably is not enough to affect the positive loop. In the genes of the negative loop (*per1b*, *per2*, and *cry1a*), in contrast, we observed a shift in the acrophases of *cry1a* and *per1b* when comparing ML and MD, with *per1b* presenting the most significant difference with an 8-h phase shift between the two groups. Although the circadian system of fish is more likely to be a multi-oscillatory system, the fish hypothalamic clock usually behaves in a similar way of the central clock in mammals, located in the SCN of the hypothalamus (López-Olmeda 2017). Thus, the clock in this tissue entrains mainly to light, with food having little influence on it (Vera et al. 2013; Costa et al. 2016). However, the responsiveness of the molecular clock in this tissue to feeding may be different depending on the fish species. For instance, a similar phase advance of the clock genes in the hypothalamus, as we have observed for *per1b*, was previously reported in goldfish fed at MD compared to ML feeding (Gómez-Boronat et al. 2018). In Wistar rats, the clock gene *per1* in the hypothalamus seems to be more influenced by feeding time than other clock genes. Restricted access to food causes a phase shift in the peak of *per1* expression in the hypothalamus (Miñana-Solis et al. 2009), altering its expression patterns (De Araujo et al. 2016). This suggests that *per1* may play a pivotal role in how feeding affects the circadian clock.

## Conclusions

This paper provides insights into the intricate interplay between the external synchronizers (light and feeding times), the circadian system and epigenetic mechanisms in the European sea bass (*Dicentrarchus labrax*). Our findings revealed that feeding time exerts differential effects on the molecular clock of the hypothalamus, causing notable shifts in the acrophases of key genes of the negative feedback loop. Interestingly, the positive loop genes exhibited no significant differences depending on feeding time. The liver, a crucial peripheral pacemaker, displayed rhythmic expression of clock genes influenced by feeding times, emphasizing the role of feeding as a potent *zeitgeber* for this organ. Moreover, this research uncovered the rhythmicity of epigenetic genes associated with methylation and demethylation processes, describing also a tight connection between feeding time and epigenetic regulation. Notably, the synchronization of circadian and epigenetic processes in the liver, with peak activity during the resting phase, highlights the potential significance of this phase for epigenetic modifications. Overall, this research contributes to our understanding of the intricate temporal dynamics governing the circadian and epigenetic landscape in the European sea bass, offering a foundation for future studies exploring the broader implications of these findings in the context of fish physiology and chronobiology.

Furthermore, this study underscores the potential implications for the aquaculture industry. Epigenetic processes are widely recognized as key mediators of environmental stimuli, like temperature (Anastasiadi et al 2017), for which the concern is rising due to the increasing global temperatures recorded each year (Atalah et al. 2024). Additionally, epigenetic processes play a key role in nutritional programming techniques, which are being actively studied to enhance fish welfare and robustness (Moghadam et al. 2015). The interplay identified between the epigenetic and circadian systems highlights the importance of factors such as feeding schedules and daily rhythms, emphasizing their role in developing more effective solutions for modern aquaculture challenges.

## Supplementary Information

Below is the link to the electronic supplementary material.

Supplementary file1 (PPTX 53 KB)

## Data Availability

Data will be made available on request.
