# Supplementary material for: Influence of feeding time on daily rhythms of locomotor activity, clock genes, and epigenetic mechanisms in the liver and hypothalamus of the European sea bass (Dicentrarchus labrax)
Source: Fish Physiol Biochem. 2025 Feb 13;51(1):50. doi: 10.1007/s10695-025-01461-7 (PMC11825647; doi:10.1007/s10695-025-01461-7)
Supplement: Supplementary file 1 — Supplementary file1 (PPTX 53 KB) [file 10695_2025_1461_MOESM1_ESM.pptx]

## Slide 1
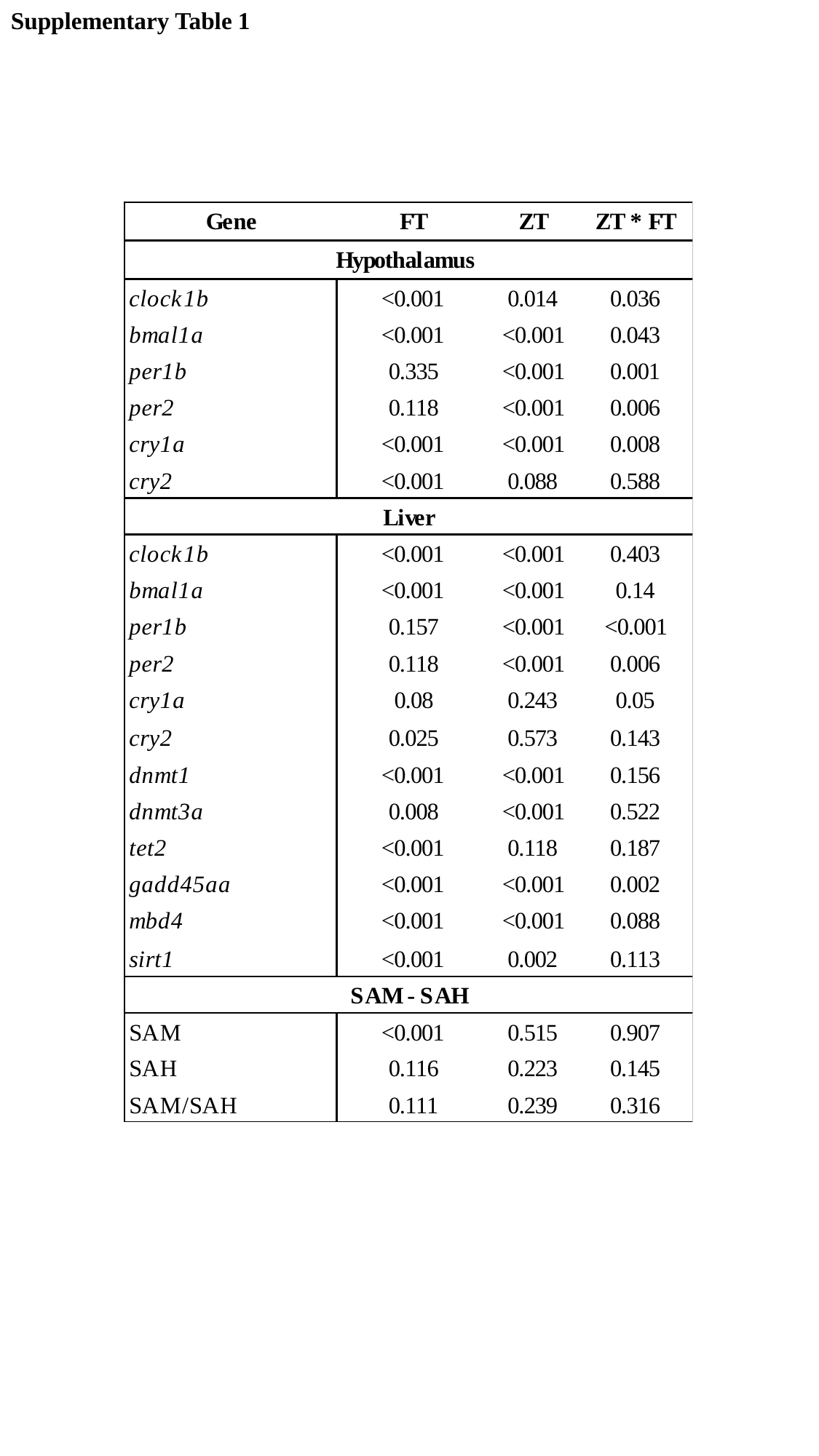

Supplementary Table 1

## Slide 2
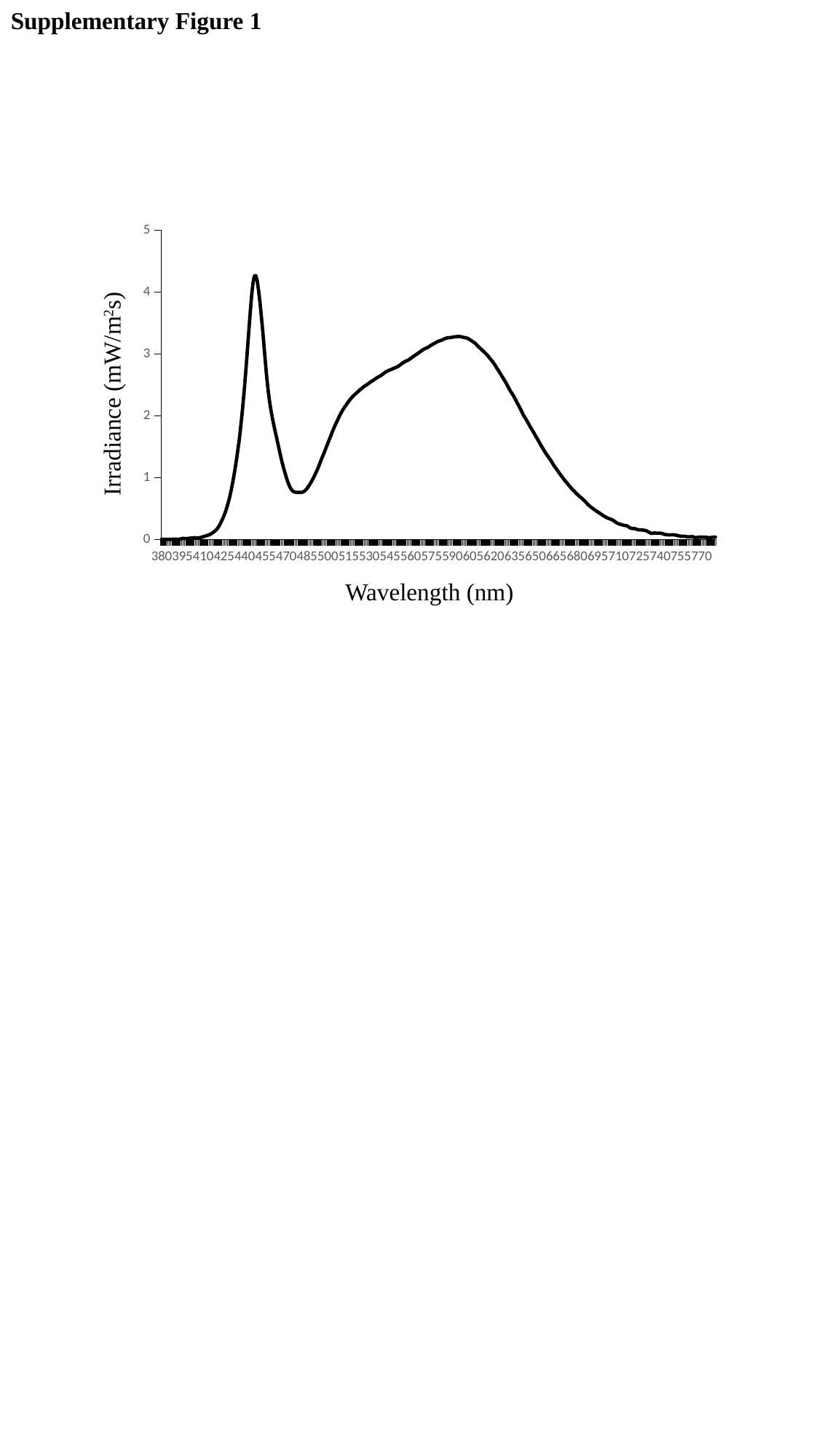

Supplementary Figure 1
### Chart
| Category | |
|---|---|
| 380 | 0.006326 |
| 381 | 0.001567 |
| 382 | 0.000685 |
| 383 | 0.001289 |
| 384 | 0.001682 |
| 385 | 0.001691 |
| 386 | 0.000712 |
| 387 | 0.000389 |
| 388 | 0.002076 |
| 389 | 0.004272 |
| 390 | 0.004418 |
| 391 | 0.003587 |
| 392 | 0.001706 |
| 393 | 0.002901 |
| 394 | 0.010078 |
| 395 | 0.015294 |
| 396 | 0.01658 |
| 397 | 0.014412 |
| 398 | 0.013529 |
| 399 | 0.016161 |
| 400 | 0.020069 |
| 401 | 0.021826 |
| 402 | 0.023447 |
| 403 | 0.025409 |
| 404 | 0.02713 |
| 405 | 0.026403 |
| 406 | 0.024857 |
| 407 | 0.026742 |
| 408 | 0.028728 |
| 409 | 0.037828 |
| 410 | 0.043584 |
| 411 | 0.052044 |
| 412 | 0.057263 |
| 413 | 0.065438 |
| 414 | 0.072588 |
| 415 | 0.081651 |
| 416 | 0.095468 |
| 417 | 0.108463 |
| 418 | 0.127825 |
| 419 | 0.145656 |
| 420 | 0.169312 |
| 421 | 0.199864 |
| 422 | 0.237592 |
| 423 | 0.282358 |
| 424 | 0.330433 |
| 425 | 0.383257 |
| 426 | 0.440593 |
| 427 | 0.508653 |
| 428 | 0.585816 |
| 429 | 0.67142 |
| 430 | 0.771486 |
| 431 | 0.880038 |
| 432 | 1.002417 |
| 433 | 1.133947 |
| 434 | 1.278738 |
| 435 | 1.440489 |
| 436 | 1.600533 |
| 437 | 1.797494 |
| 438 | 2.009078 |
| 439 | 2.247283 |
| 440 | 2.504177 |
| 441 | 2.779968 |
| 442 | 3.075344 |
| 443 | 3.373791 |
| 444 | 3.67697 |
| 445 | 3.944787 |
| 446 | 4.148668 |
| 447 | 4.25845 |
| 448 | 4.267372 |
| 449 | 4.18435 |
| 450 | 4.029387 |
| 451 | 3.840854 |
| 452 | 3.616664 |
| 453 | 3.374998 |
| 454 | 3.110827 |
| 455 | 2.84787 |
| 456 | 2.60564 |
| 457 | 2.397357 |
| 458 | 2.222474 |
| 459 | 2.081414 |
| 460 | 1.960505 |
| 461 | 1.852602 |
| 462 | 1.749256 |
| 463 | 1.646694 |
| 464 | 1.544603 |
| 465 | 1.443163 |
| 466 | 1.343643 |
| 467 | 1.247757 |
| 468 | 1.158211 |
| 469 | 1.084763 |
| 470 | 1.006946 |
| 471 | 0.939057 |
| 472 | 0.880381 |
| 473 | 0.832274 |
| 474 | 0.799195 |
| 475 | 0.777287 |
| 476 | 0.766756 |
| 477 | 0.762711 |
| 478 | 0.759549 |
| 479 | 0.759482 |
| 480 | 0.758764 |
| 481 | 0.759827 |
| 482 | 0.767313 |
| 483 | 0.778516 |
| 484 | 0.79816 |
| 485 | 0.822288 |
| 486 | 0.854577 |
| 487 | 0.88941 |
| 488 | 0.928698 |
| 489 | 0.968398 |
| 490 | 1.010285 |
| 491 | 1.05764 |
| 492 | 1.106135 |
| 493 | 1.157001 |
| 494 | 1.214584 |
| 495 | 1.272063 |
| 496 | 1.329537 |
| 497 | 1.381413 |
| 498 | 1.436767 |
| 499 | 1.495205 |
| 500 | 1.551437 |
| 501 | 1.608343 |
| 502 | 1.664729 |
| 503 | 1.724055 |
| 504 | 1.77948 |
| 505 | 1.829373 |
| 506 | 1.879282 |
| 507 | 1.926036 |
| 508 | 1.97513 |
| 509 | 2.021398 |
| 510 | 2.060843 |
| 511 | 2.099206 |
| 512 | 2.13396 |
| 513 | 2.164463 |
| 514 | 2.199001 |
| 515 | 2.228019 |
| 516 | 2.259489 |
| 517 | 2.285974 |
| 518 | 2.310792 |
| 519 | 2.332661 |
| 520 | 2.353584 |
| 521 | 2.37387 |
| 522 | 2.394671 |
| 523 | 2.416295 |
| 524 | 2.432418 |
| 525 | 2.452728 |
| 526 | 2.469732 |
| 527 | 2.486557 |
| 528 | 2.499347 |
| 529 | 2.513385 |
| 530 | 2.530872 |
| 531 | 2.547828 |
| 532 | 2.562499 |
| 533 | 2.577204 |
| 534 | 2.592365 |
| 535 | 2.607172 |
| 536 | 2.620574 |
| 537 | 2.633113 |
| 538 | 2.644593 |
| 539 | 2.662214 |
| 540 | 2.677949 |
| 541 | 2.695506 |
| 542 | 2.710049 |
| 543 | 2.721647 |
| 544 | 2.732057 |
| 545 | 2.741989 |
| 546 | 2.750657 |
| 547 | 2.760895 |
| 548 | 2.77015 |
| 549 | 2.779792 |
| 550 | 2.788732 |
| 551 | 2.802354 |
| 552 | 2.817256 |
| 553 | 2.835084 |
| 554 | 2.851214 |
| 555 | 2.86578 |
| 556 | 2.878657 |
| 557 | 2.889244 |
| 558 | 2.899014 |
| 559 | 2.912585 |
| 560 | 2.928079 |
| 561 | 2.947153 |
| 562 | 2.962721 |
| 563 | 2.976917 |
| 564 | 2.992512 |
| 565 | 3.007939 |
| 566 | 3.023353 |
| 567 | 3.040132 |
| 568 | 3.056403 |
| 569 | 3.072103 |
| 570 | 3.083764 |
| 571 | 3.093247 |
| 572 | 3.102957 |
| 573 | 3.114588 |
| 574 | 3.131248 |
| 575 | 3.145468 |
| 576 | 3.158196 |
| 577 | 3.169999 |
| 578 | 3.182383 |
| 579 | 3.195478 |
| 580 | 3.205799 |
| 581 | 3.213148 |
| 582 | 3.219813 |
| 583 | 3.22919 |
| 584 | 3.241156 |
| 585 | 3.251635 |
| 586 | 3.257986 |
| 587 | 3.261723 |
| 588 | 3.262925 |
| 589 | 3.265638 |
| 590 | 3.270406 |
| 591 | 3.274762 |
| 592 | 3.276958 |
| 593 | 3.27929 |
| 594 | 3.282366 |
| 595 | 3.281966 |
| 596 | 3.279086 |
| 597 | 3.272995 |
| 598 | 3.268343 |
| 599 | 3.263999 |
| 600 | 3.258898 |
| 601 | 3.252891 |
| 602 | 3.239805 |
| 603 | 3.224784 |
| 604 | 3.210874 |
| 605 | 3.19464 |
| 606 | 3.18053 |
| 607 | 3.159431 |
| 608 | 3.135435 |
| 609 | 3.113602 |
| 610 | 3.092127 |
| 611 | 3.071446 |
| 612 | 3.050158 |
| 613 | 3.032115 |
| 614 | 3.010173 |
| 615 | 2.984896 |
| 616 | 2.960344 |
| 617 | 2.929811 |
| 618 | 2.905052 |
| 619 | 2.876747 |
| 620 | 2.845386 |
| 621 | 2.813294 |
| 622 | 2.774597 |
| 623 | 2.74346 |
| 624 | 2.705994 |
| 625 | 2.67012 |
| 626 | 2.632457 |
| 627 | 2.596597 |
| 628 | 2.560555 |
| 629 | 2.521804 |
| 630 | 2.478409 |
| 631 | 2.437351 |
| 632 | 2.398074 |
| 633 | 2.364218 |
| 634 | 2.327905 |
| 635 | 2.287097 |
| 636 | 2.243631 |
| 637 | 2.199858 |
| 638 | 2.161598 |
| 639 | 2.11627 |
| 640 | 2.070451 |
| 641 | 2.024469 |
| 642 | 1.985813 |
| 643 | 1.952449 |
| 644 | 1.91209 |
| 645 | 1.872361 |
| 646 | 1.831337 |
| 647 | 1.794906 |
| 648 | 1.758877 |
| 649 | 1.717465 |
| 650 | 1.676749 |
| 651 | 1.640999 |
| 652 | 1.60079 |
| 653 | 1.561404 |
| 654 | 1.520599 |
| 655 | 1.484263 |
| 656 | 1.448017 |
| 657 | 1.410664 |
| 658 | 1.376336 |
| 659 | 1.34453 |
| 660 | 1.313263 |
| 661 | 1.28339 |
| 662 | 1.244676 |
| 663 | 1.207418 |
| 664 | 1.174427 |
| 665 | 1.143185 |
| 666 | 1.112653 |
| 667 | 1.07982 |
| 668 | 1.049647 |
| 669 | 1.020687 |
| 670 | 0.990666 |
| 671 | 0.960379 |
| 672 | 0.933259 |
| 673 | 0.907333 |
| 674 | 0.878876 |
| 675 | 0.852458 |
| 676 | 0.82618 |
| 677 | 0.802915 |
| 678 | 0.780828 |
| 679 | 0.75556 |
| 680 | 0.733298 |
| 681 | 0.71357 |
| 682 | 0.690938 |
| 683 | 0.6746 |
| 684 | 0.655283 |
| 685 | 0.631814 |
| 686 | 0.613553 |
| 687 | 0.588035 |
| 688 | 0.562712 |
| 689 | 0.542719 |
| 690 | 0.524361 |
| 691 | 0.507916 |
| 692 | 0.49251 |
| 693 | 0.47413 |
| 694 | 0.45945 |
| 695 | 0.445333 |
| 696 | 0.430145 |
| 697 | 0.415895 |
| 698 | 0.400522 |
| 699 | 0.383299 |
| 700 | 0.371282 |
| 701 | 0.359705 |
| 702 | 0.346554 |
| 703 | 0.338556 |
| 704 | 0.329648 |
| 705 | 0.321869 |
| 706 | 0.30952 |
| 707 | 0.29567 |
| 708 | 0.278567 |
| 709 | 0.26333 |
| 710 | 0.254878 |
| 711 | 0.245335 |
| 712 | 0.2415 |
| 713 | 0.233762 |
| 714 | 0.227312 |
| 715 | 0.225102 |
| 716 | 0.219686 |
| 717 | 0.208371 |
| 718 | 0.190775 |
| 719 | 0.180406 |
| 720 | 0.17684 |
| 721 | 0.177733 |
| 722 | 0.172873 |
| 723 | 0.164634 |
| 724 | 0.158001 |
| 725 | 0.154279 |
| 726 | 0.153616 |
| 727 | 0.152204 |
| 728 | 0.150717 |
| 729 | 0.145301 |
| 730 | 0.141247 |
| 731 | 0.131045 |
| 732 | 0.11609 |
| 733 | 0.103899 |
| 734 | 0.097896 |
| 735 | 0.101188 |
| 736 | 0.104307 |
| 737 | 0.103145 |
| 738 | 0.099972 |
| 739 | 0.100491 |
| 740 | 0.103176 |
| 741 | 0.097908 |
| 742 | 0.091541 |
| 743 | 0.08298 |
| 744 | 0.078596 |
| 745 | 0.076038 |
| 746 | 0.074255 |
| 747 | 0.072584 |
| 748 | 0.073746 |
| 749 | 0.07671 |
| 750 | 0.073668 |
| 751 | 0.071249 |
| 752 | 0.067562 |
| 753 | 0.059215 |
| 754 | 0.057109 |
| 755 | 0.051323 |
| 756 | 0.049462 |
| 757 | 0.051001 |
| 758 | 0.04962 |
| 759 | 0.047102 |
| 760 | 0.039315 |
| 761 | 0.043405 |
| 762 | 0.042615 |
| 763 | 0.045785 |
| 764 | 0.041034 |
| 765 | 0.031583 |
| 766 | 0.031186 |
| 767 | 0.033304 |
| 768 | 0.036711 |
| 769 | 0.03713 |
| 770 | 0.036505 |
| 771 | 0.038228 |
| 772 | 0.036739 |
| 773 | 0.035842 |
| 774 | 0.031777 |
| 775 | 0.029649 |
| 776 | 0.028167 |
| 777 | 0.031582 |
| 778 | 0.035981 |
| 779 | 0.035519 |
| 780 | 0.03858 |Irradiance (mW/m2s)
Wavelength (nm)

## Slide 3
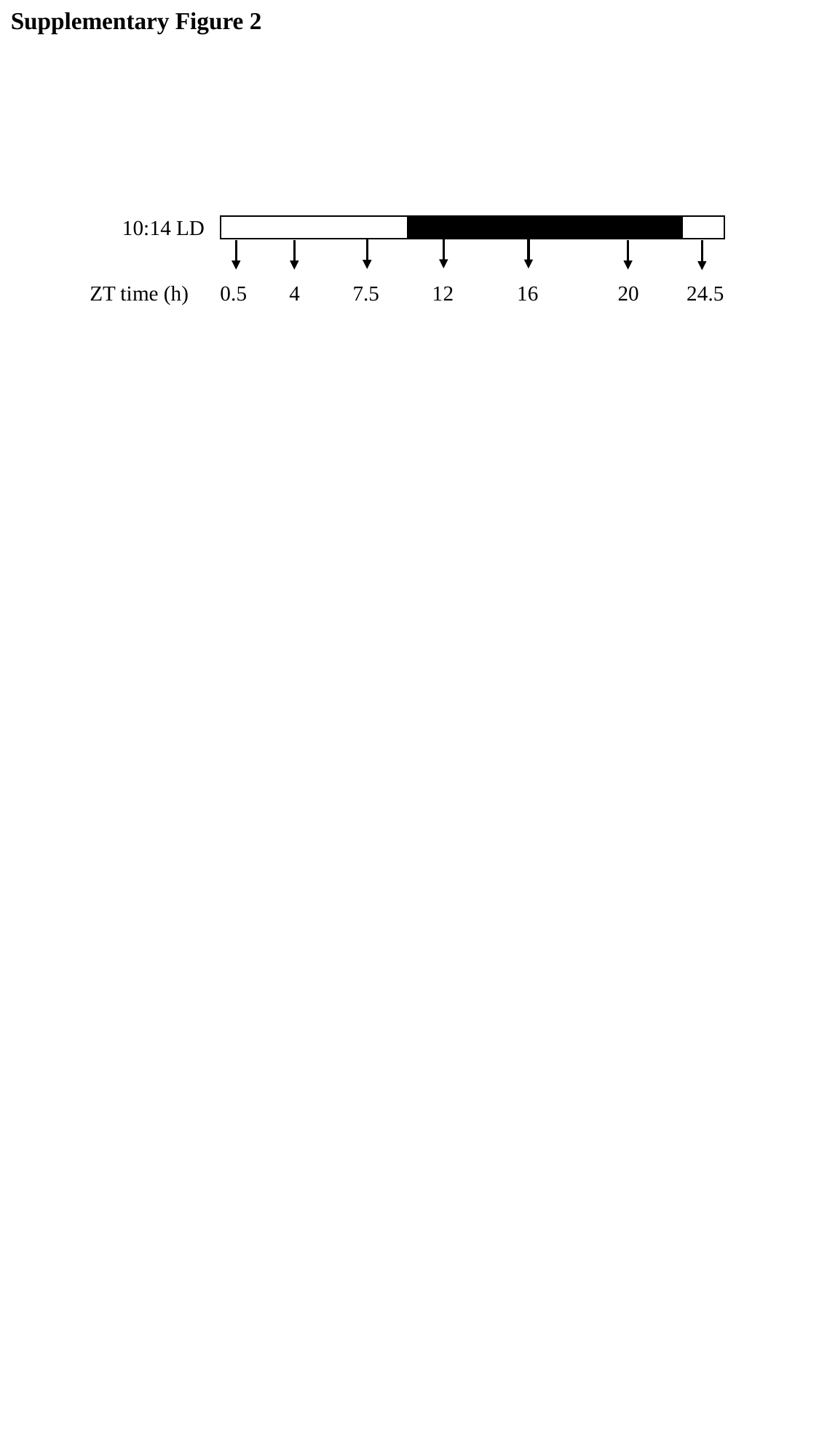

Supplementary Figure 2
10:14 LD
ZT time (h)
 0.5 4 7.5 12 16 20 24.5
